# Early postoperative pulmonary complications following heart transplantation

**DOI:** 10.1186/cc14350

**Published:** 2015-03-16

**Authors:** A Camkiran Firat, Ö Kömürcü, P Zeyneloglu, M Türker, A Sezgin, A Pirat

**Affiliations:** 1 Baskent University, Ankara, Turkey

## Introduction

The aim of this study was to determine the types, incidence, and risk factors for early postoperative pulmonary complications in heart transplantation recipients.

## Methods

We retrospectively collected data from the records of consecutive heart transplantations from January 2003 to December 2013. A total of 83 patients underwent heart transplantation. Those patients younger than 10 years (*n *= 9) and the patients who died intraoperatively (*n *= 1) or during the first postoperative day (*n *= 1) were not included in the analyses. The data collected for each case were demographic features, duration of mechanical ventilation, respiratory problems that developed during the ICU stay, and early postoperative mortality (<30 days). The respiratory complications that we sought were pleural effusion, pneumonia, pulmonary atelectasis, pulmonary edema, pneumothorax, and acute respiratory failure.

## Results

Of the 72 patients considered, 52 (72.2%) were male. The mean age at the time of transplantation was 32.1 ± 16.6 years. The mean duration of postoperative mechanical ventilation was 71.8 ± 126.6 hours. The mean length of ICU stay was 13.5 ± 18.0 days. Two patients (2.8%) and one patient (1.4%) required extracorporeal membrane oxygenation support and intra-aortic balloon pump support, respectively, due to low cardiac output or primary graft failure postoperatively. Twenty-five patients (34.7%) developed early postoperative respiratory complications. The most frequent problem was pleural effusion (*n *= 19, 26.4%) followed by atelectasis (*n *= 6, 8.3%), acute respiratory distress syndrome (*n *= 5, 6.9%), pulmonary edema (*n *= 4, 5.6%), and pneumonia (*n *= 3, 4.2%). Postoperative duration of mechanical ventilation (44.2 ± 59.2 hours vs. 123.8 ± 190.8 hours, *P *= 0.005) and the length of ICU stay postoperatively (10.1 ± 5.8 hours vs. 19.8 ± 28.9 hours, *P *= 0.03) were longer among patients who had respiratory problems. Postoperative length of stay in the hospital (22.3 ± 12.5 days vs. 30.3 ± 38.3 days, *P *= 0.75) was similar in the two groups. The overall mortality rate was 12.5% (*n *= 9 patients). The patients who had respiratory problems did not show higher mortality than those who did not have respiratory problems (16.0% vs. 10.6%, *P *= 0.71).

## Conclusion

Respiratory complications were relatively common in our cohort of heart transplant recipients. However, these complications were mostly self-limiting and did not result in increased mortality.

